# Social Media Use for Health Purposes: Systematic Review

**DOI:** 10.2196/17917

**Published:** 2021-05-12

**Authors:** Junhan Chen, Yuan Wang

**Affiliations:** 1 Department of Communication University of Maryland College Park, MD United States

**Keywords:** social media, health communication, health researchers, health practitioners, health institutions, systematic review

## Abstract

**Background:**

Social media has been widely used for health-related purposes, especially during the COVID-19 pandemic. Previous reviews have summarized social media uses for a specific health purpose such as health interventions, health campaigns, medical education, and disease outbreak surveillance. The most recent comprehensive review of social media uses for health purposes, however, was conducted in 2013. A systematic review that covers various health purposes is needed to reveal the new usages and research gaps that emerge in recent years.

**Objective:**

This study aimed to provide a systematic review of social media uses for health purposes that have been identified in previous studies.

**Methods:**

The researchers searched for peer-reviewed journal articles published between 2006 and 2020 in 12 databases covering medicine, public health, and social science. After coding the articles in terms of publication year, journal area, country, method, social media platform, and social media use for health purposes, the researchers provided a review of social media use for health purposes identified in these articles.

**Results:**

This study summarized 10 social media uses for various health purposes by health institutions, health researchers and practitioners, and the public.

**Conclusions:**

Social media can be used for various health purposes. Several new usages have emerged since 2013 including advancing health research and practice, social mobilization, and facilitating offline health-related services and events. Research gaps exist regarding advancing strategic use of social media based on audience segmentation, evaluating the impact of social media in health interventions, understanding the impact of health identity development, and addressing privacy concerns.

## Introduction

Social media has been widely used in health contexts by various users. For individuals, social media is a primary source of COVID-19 information for patients [1]. Social media is used by 80% of cancer patients to connect with peers [2]. For health organizations, more than 80% of US state health departments have social media accounts [3]. Among health professionals, 65% of radiologists across the United States and Europe use social media for various health-related reasons [4]. A review of the state of the art can provide guidance for practitioners who intend to use social media and for researchers who intend to advance our understanding of social media use for health purposes.

Before reviewing social media use for health purposes, it is necessary to outline the scope of social media and health purposes. Social media has been defined in different ways. Some definitions focus on the technological features of social media that distinguish it from traditional technologies. For example, Kaplan and Haenlein [5] emphasized that social media is a type of application based on the internet and web 2.0 technology. Other definitions focus on the communication features of social media that distinguish it from traditional media. For example, McGowan et al [6] defined social media as an online environment where users can contribute to the content and consume content mostly generated by other users. They also emphasized that content being “created by users for users” is an important feature that distinguishes social media from traditional media [6]. From a communication perspective, this paper emphasizes the communication features of social media and considers social media as a web 2.0–based platform for individuals to get access to, share, and generate content. Health purposes include health-related goals such as health promotion, medical service and administration, health research, medical education and training, and health-related social movements. This review focuses on users purposively getting access to, sharing, and generating content on social media in order to achieve various health purposes.

Previous reviews about social media use for health mostly focused on using social media for a single health purpose. These reviews summarized social media as a tool for health interventions [7], health campaigns [8], medical education [9], disease outbreak surveillance [10], and health promotion and behavior change [11]. The most recent comprehensive review of social media use for various health-related purposes was conducted by Moorhead et al [12] and included studies published between 2002 and 2012. However, multiple new uses have emerged between 2013 and 2020 given the rapid development of social media and the need for effective communication and infodemiology practice in the face of emerging health risks. Following Moorhead et al [12], this study reviewed articles published between 2006 and 2020 and categorized social media uses for health purposes in terms of user types, namely, health institutions, health researchers and professionals, and the public. By conducting a systematic review, we aimed to summarize social media use for health purposes, identify new uses that have emerged since 2013 as compared with Moorhead et al [12], and discuss research gaps and future directions for research in social media use for health purposes.

## Methods

### Searching Strategy and Inclusion Criteria

**Figure 1:**
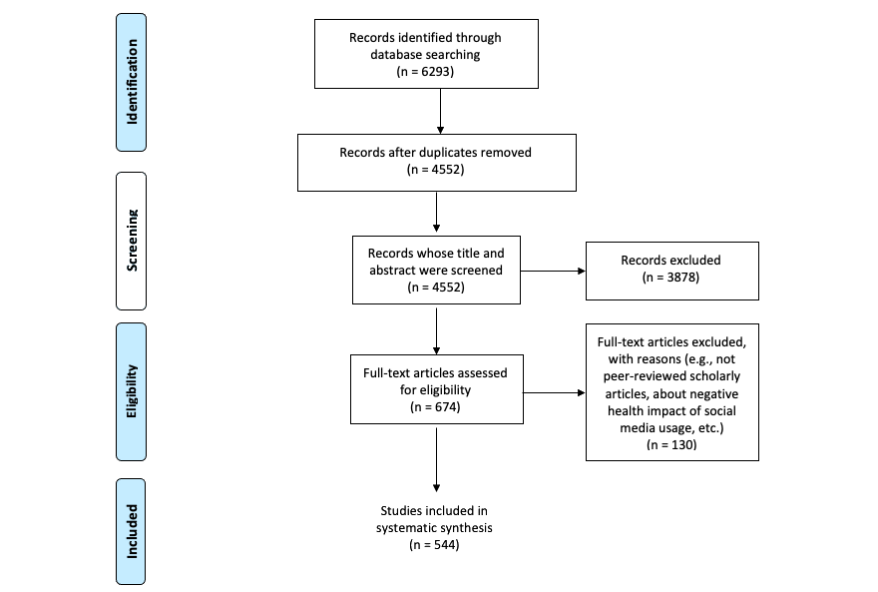
Article search and screening process.

This review followed the PRISMA (Preferred Reporting Items for Systematic Reviews and Meta-analyses) guideline [13] to search and screen articles as shown in Figure 1. In the first step, the researchers searched 12 databases through ProQuest and EBSCO, including important databases in public health, medical, and social science areas such as MEDLINE, Academic Search Complete, PsycINFO, CINAHL, Psychology and Behavioral Science Collection, and Coronavirus Research Database. The search string was “AB(health) AND AB(“social media” OR “social network” OR Facebook OR LinkedIn OR Twitter OR Instagram OR Pinterest OR WeChat OR Weibo).” Additional restrictions (ie, published between 2006 and 2020, peer-reviewed scholarly journal article, full text available, and written in English) were added to the search requirement. The time range was set from 2006 to the end of 2020 because most of the current popular social media platforms such as Twitter and Facebook have been created or open to the public since 2006. The initial search retrieved 6293 articles.

In the next step, the two researchers screened items retrieved from the databases. An article was included if the study identifies at least one social media use for health purposes (eg, a study that finds that parents use social media to seek information about breastfeeding), examines the impact of using social media for health purposes (eg, a study that examines the effectiveness of a social media–based health intervention), or explores how to use social media for health purposes (eg, a study that examines how to detect mental health risk through analyzing social media posts). After reviewing the titles, abstracts, and full text, the researchers included 544 articles in the final sample. Articles were excluded for reasons such as not being scholarly journal articles, focusing on the negative health impacts of using social media such as depression and obesity, describing social media networks or social media post characteristics without discussing how to intentionally use social media for health purposes, examining health promotion within an offline social network, or examining social media use in general context rather than health context.

### Coding Process

The preliminary coding scheme was developed by one researcher based on an initial review of 20% of the articles included. The two researchers coded 20 articles using the preliminary coding scheme and met to discuss the applicability of the preliminary coding categories and possible additional coding categories. The coding scheme was finalized after the two researchers reached an agreement after the discussion. The finalized coding scheme included 8 dimensions: publication year, journal area (eg, public health and medical area, communication, psychology), the country where the study was conducted (eg, United States, United Kingdom, China), type of article (eg, empirical studies, review paper, protocol), methodology used in empirical studies (eg, experiment, survey, interview), type of social media users (ie, health institution, health researcher and professional, publics), and social media use for health purposes. Social media use for health purposes were grouped under social media users. There are 4 usages by health institutions (ie, infoveillance, disseminate health information and combat misinformation, health intervention, and social mobilization), 3 usages by health researchers and professionals (ie, health-related research, professional development, and facilitate doctor-patient communication and offline services), and 3 usages by the public (ie, seek and share health information, exchange social support in an online community, and track and share one’s health statuses or activities). The intercoder reliability indicated by Krippendorff alpha ranged from a low of 0.86 to a high of 1.00. The reliability is satisfactory compared with the 0.80 criterion [14].

## Results

### Characteristics of the Studies

All articles were published between 2016 and 2020. There were 25 articles published in 2016, 42 in 2017, 28 in 2018, 185 in 2019, and 264 in 2020. The number of publications continuously increased during the 5 years, showing that social media use for health purposes received growing attention from researchers.

Regarding methodology, most of the articles were empirical studies (n=399). Among the empirical studies, 294 used a quantitative method including experiment, survey, content analysis, and network analysis, and 74 studies used a qualitative method including interview, focus group, and discourse/thematic analysis. There were 15 studies using a mixed method and 16 studies used other methods such as data mining and describing social media metrics of campaign messages (eg, number of likes and views). The rest of the articles were review papers (n=78), commentaries (n=37), protocols (n=22), and theoretical and methodological papers (n=8).

Regarding fields of publication, most articles were published in journals in medical and public health (n=489) followed by journals in communication (n=26), psychology (n=7), sociology (n=6), information science (n=5), interdisciplinary journals (n=5), and journals of other fields (n=6) such as technology, computing, and behavior research method.

Most of the articles featured social media use in the United States (n=170) followed by China (n=64), United Kingdom (n=20), Australia (n=14), Canada (n=7), South Africa (n=6), Iran (n=5), Malaysia (n=4), Ghana (n=4), and India (n=4). A total of 43 articles featured social media use in other countries such as Jordan, South Korea, Denmark, and Sweden. Some studies featured social media use in more than one country or internationally (n=27). There were 176 studies that did not specify the geographic scope of social media use. For example, there were studies that analyzed all the English written posts related to a disease on social media or reviewed all the relevant literature about social media–based health intervention.

Regarding the social media platforms used for health purposes, most studies (n=231) examined social media in general without specifying use on certain social media platforms. Other studies featured Twitter (n=79) followed by Facebook (n=76), WeChat (n=33), online forums (n=16), Sina Weibo (n=13), Reddit (n=8), YouTube (n=8), WhatsApp (n=6), Instagram (n=6), and other platforms such as Pinterest, Yelp, and Yahoo! Answer (n=10). There were also 58 studies that featured more than one social media platform.

### Social Media Use for Health Purposes

This review summarized 10 uses of social media for health purposes. Uses were grouped into 3 categories according to the type of social media users, namely, health institution, health researcher and professional, and the public. The following sections summarize social media uses by each type of user and the benefits and challenges related to each use.

#### Health Institution

Health institutions include government health agencies and nongovernmental health organizations such as the World Health Organization. The review shows that health institutions mainly used social media for infoveillance (n=94), disseminating health information and combating misinformation (n=76), health intervention (n=168), and social mobilization (n=7).

##### Infoveillance

Infoveillance is the application of infodemiology with a primary aim of surveillance, which refers to surveilling and analyzing the unstructured information available on the internet in order to inform public health and public policy [15]. In this review, we focused on the surveillance of information available on social media in order to inform public health. Specifically, articles in this category analyzed social media data to (1) surveil potential public health risks including the onset of individual illnesses and disease outbreaks in a population (n=70) and (2) monitor public responses to health issues (n=25).

First, social media data have been analyzed to predict the future illness onset for individual users. For example, the language used in users’ posts such as life stress and anxiety can predict future occurrence of mental illness and distinguish different types of mental illness [16]. In addition, special characters used in social media posts such as emojis can also help identify illness syndromes such as asthma and difficulty breathing syndrome [17]. The use of social media to detect individual illness is especially beneficial for people who are at risk but less motivated to seek diagnosis and treatment; social media surveillance can supplement the traditional doctor-patient interaction method for illness detection [17] and can be combined with social media–based interventions to proactively provide health advice to those who are less likely to seek help [18].

Second, social media data can be analyzed to predict infectious disease outbreaks among a population such as in the COVID-19 outbreak [19]. The studies showed that social media data can provide an accurate prediction of disease outbreak case count [19]. In addition to outbreak prediction, demographic and geographic data obtained from social media can inform medical research and practice of the characteristics of people who are at risk of being infected [20]. Consistent with a recent systematic review of social media–based surveillance systems for health care [21], this study found that Twitter was the most used social media platform for individual illness and disease outbreak surveillance.

Third, user-generated social media posts can provide insights about the public’s cognitive and behavioral responses to health-related issues. Studies have analyzed social media posts to understand public concerns and sentiments about ongoing disease outbreaks such as Zika [22] and COVID-19 [23], investigated public attitudes toward and actual implementation of recommended disease prevention actions [20,24], monitored public discussion about controversial health topics such as vaccination [25] and e-cigarettes [26], and gauged public support of health-related policies [27]. Assessing social media data to understand public responses to health issues not only yields results comparable to national survey data but also provides insights for health-related theoretical models [25]. More importantly, traditional public surveys may take weeks and are expensive, while social media provides real-time and inexpensive data to evaluate the effectiveness of public health communication, which allows communication practitioners to adapt timely communication strategies to public needs [23].

##### Disseminate Health Information and Combat Misinformation

With the ability to disseminate information quickly and widely, social media can be used by health institutions to post health information and share information with the public. Health institutions can inform the public about common health topics such as healthy living, immunization, and smoking [7]; communicate the risk of disease outbreaks [28]; quickly provide instructions about prevention behaviors to a broad audience during disease outbreaks [29]; and share the latest news and inform the public of the government’s handling of disease outbreaks [28,30]. Research efforts have been devoted to exploring strategies for generating health content on social media and increasing retransmission [31,32].

Social media has several advantages over other media outlets when used for disseminating health information. First, social media is seen as the fastest among available channels to share alerts and updates about disease outbreaks [28]. Second, social media allows leveraging various media forms to engage the public. For example, by integrating hyperlinks, social media posts can direct the public to other online resources for additional health information [28,33]. Health institutions can also share podcast audios and YouTube videos on various social media platforms to deliver health information [34,35]. Third, social media can provide a communication platform for stakeholders during disease outbreaks. Government social media accounts may become official information sources that provide content about disease outbreaks for local agencies and journalists in a timely manner [28,33].

With the increased volume of misinformation during the COVID-19 pandemic, many studies focused on the concern of rapid misinformation transmission on social media and proposed approaches to combat misinformation through social media. For example, researchers suggested health institutions increase efforts to monitor and enforce fact-checking of dubious information on social media [36,37], promote strategic and timely refutation to misinformation on social media [38], share personal experiences on social media to refute rumors [39], and use search optimization strategies within the social media platforms to redirect users who ask health-related questions to reliable information sources [40]. In addition to combating misinformation through social media, health institutions may also encourage health professionals to establish interpersonal relationships with patients and invite patients to conversations about misinformation that they may encounter online [41]. Although the effectiveness of the approaches proposed by articles in this review have not been formally tested, there have been many studies testing the effectiveness of different message design features in correcting misinformation. For example, Gesser-Edelsburg and colleagues [42] found that a theory-based correction message posted on Facebook by health authorities was more effective in correcting vaccination misinformation than a common correction message. Practitioners may design and post misinformation-correcting messages on social media based on the strategies suggested by these message design studies.

##### Health Intervention

Social media has been widely integrated into health interventions and can perform multiple functions. First, social media was used to offer health resources including delivering health information to audiences [43], motivating participation in health-related events [44], and directing campaign audiences and intervention participants to other health resources [45,46]. Second, social media was used to support interaction with audience including engaging campaign audiences in 2-way communication with the institutions and health professionals [47,48] and providing platforms for peer support group discussion [49]. Third, social media was used to motivate health behavior change; intervention participants used social media to document and share their own progress of health behavior change such as uploading a picture of an everyday healthy meal [50] and sharing virtual awards of weekly achievement in physical activities [51], setting group challenges toward health goals [52], and engaging in health behavior competition with peers [53]. Last, at the preparing stage of an intervention, social media was also used to reach broader and more varied populations, including hard-to-reach populations [54,55].

Research effort has also focused on improving the design and evaluation of social media–based health interventions. Studies have examined approaches to evaluating campaign outcomes [56], proposed theoretical frameworks for effective health interventions design [57], explored social media user segments to inform intervention development [58], and examined user acceptance and suggestions of receiving social media–based interventions [59]. In general, empirical evidence shows that integrating social media in health interventions is accepted by target populations [59] and effective in increasing health knowledge, reducing risky behaviors, and adopting health behaviors [60]. Researchers have also discussed challenges and raises concerns regarding social media–based health intervention; for example, researchers must rigorously evaluate social media–based interventions throughout the stages of development and implementation, involve populations experiencing health disparities in the studies, and address privacy concerns [61].

##### Social Mobilization

Since social media can reach varied stakeholders and a wide audience in society, nongovernmental health organizations can use social media to mobilize social resources. Studies found that these organizations leveraged social media to advocate for change in public policies related to health issues such as HIV/AIDS [62] and mental health [63], raise funds for individual medical care and health-related research [64,65], and raise awareness and promote actions to address health problems such as medical equipment shortages [66]. Although a formal test of the effectiveness of using social media for social mobilization is lacking, the successful cases described in the articles show that social media provides a platform for nongovernmental health organizations to effectively mobilize resources and advocate for collective actions to achieve their goals [62].

#### Health Researchers and Practitioners

Social media use by health researchers includes facilitating health-related research (n=45), professional development (n=34), and doctor-patient communication and offline services (n=36).

##### Facilitate Health-Related Research

Social media can facilitate health-related research in two ways: provide additional data to learn about patients’ disease experience and recruit research participants. First, by analyzing patients’ conversations on social media, researchers can gauge patients’ understanding of the disease and their coping strategies [67], identify their concerns about the disease [68], understand their barriers to health behavior change [69], identify symptoms related to the disease [70], and assess patients’ experience after recovery [71]. Because patient self-reported illness experience is not routinely reported to and recorded by physicians, analyzing patient discussions on social media may enhance health researchers’ and professionals’ understanding of patient experiences beyond what can be learned from traditional sources of health data [72].

Second, social media can help researchers recruit participants for health-related research. Studies found that social media performs as well as or even better than traditional recruiting methods in terms of cost and number of participants enrolled [73]. Social media is also useful in recruiting hard-to-reach populations such as immigrants [74]. Social media recruitment can generate a representative sample; a study comparing a social media–recruited sample with the underlying population found that the sample was representative in 8 of the 13 characteristics studied including demographics and health-related factors [75].

##### Professional Development

Health professionals and researchers may use social media for their own professional development such as learning, collaboration, and career advancement. Social media can be used to collaborate on research projects and practices [76], access and share trending research findings and medical knowledge [77,78], broaden their exposure to funders and publishers [79], conduct a job search [80], follow medical conferences remotely [81], market their team and services, and discuss interesting or difficult cases with colleagues [4]. Studies found that different social media platforms serve different roles in the professional development of health professionals and researchers; Pinterest was mostly used for health care–quality education [82], Twitter for gathering news and information on conferences, and LinkedIn for career advancement [81]. Nearly 85% of health professionals agreed that social media can be an effective tool for educational purposes [82], and 71% of health professionals, researchers, and businesspeople in the urology discipline agreed that social media is useful for career development [83].

##### Facilitate Doctor-Patient Communication and Offline Health Services

Health professionals use social media for doctor-patient communication including responding to questions posted by patients [84], offering online consultation [85], and proactively providing advice and health information to social media followers [86]. In addition, social media can be used to support offline health services. Health professionals can use social media to inform patients of the results of their examination [4], encourage compliance with medication [87], receive feedback from patients about their health services [4], and collect information from patients after discharge to inform future practices [88]. Encouraging interactions between health practitioners and patients on social media may benefit both patients’ well-being and patient-physician relationships [89]. In addition, social media can also be used to provide medical services such as appointments, medical inquiries, personal information management, and medical charge payment, which can increase the accessibility of medical care and improve patient experiences [90].

However, increasing use of social media by health professionals and researchers is accompanied by concerns and challenges. Many studies raised concerns about risks to patients’ privacy and confidentiality [80] and health professionals lacking knowledge about social media [4]. Studies also discussed challenges such as protecting content generators’ intellectual property [91] and managing negative comments from patients [4]. Most of these articles called for developing a guideline or a regulation regarding ethical, legal, and technological issues [4,80] and providing training for health professionals and researchers on the proper use of social media [91,92].

#### Public

The public includes healthy people and those with health concerns, including patients with known diagnoses and populations at risk of certain health problems, such as men who have sex with men, first-time pregnant mothers, and LGBTQIA+ (lesbian, gay, bisexual, transgender, queer, intersex, asexual) youth. The reviews show that the public uses social media to seek and share health-related information (n=108), exchange social support in online communities (n=95), and track and share health statuses or activities (n=10). 

##### Seek and Share Health-Related Information

Seeking and sharing health information is the most common use by the public. The public uses social media to follow and share news about trending health issues such as flu [93] and COVID-19 [1], find information on daily health behaviors such as fitness and healthy food choices [94-96], search for health advice for their own health concern such as pregnancy-related information [97], request a crowd diagnosis or second opinion after seeing a health care professional about their illness [98], access health care law [70], follow social media accounts of health organizations and professionals [99,100], and learn about physicians and hospitals to inform their choices [101]. Social media has become a primary information source for the public with varying information needs [1,102].

However, many studies raised concerns of social media information quality and its impact on individual health outcomes [103]. Indeed, empirical studies provided grounds for the concern. Sallam et al [104] found that people who relied on social media for health information had a lower COVID-19 knowledge score compared with those who relied on doctors and scientific journals for information. In addition, people who sought information on social media did not confirm its accuracy with health professionals, and health professionals disagreed with the information found on social media on 36.7% of occasions when people checked the information accuracy with health professionals [105]. This evidence calls for efforts to reduce misinformation on social media and improve the ability of the public to find reliable information sources on social media.

##### Exchange Social Support in Online Communities

Social support includes informational support, emotional support, esteem support, network support, and tangible support [106]. Except for tangible support, other types of social support were widely found in interactions in the online health community.

People with certain health concerns can receive and provide informational support content. They may read the experience of others diagnosed with the same illness, share details of their own medication and diagnosis [107,108], share and access medical knowledge such as treatment options and self-care activities [109], learn about health laws [110], and solicit recommendations of doctors and hospitals [99]. They may also proactively request health information and advice [111] and validate health information with others [112].

Emotional and esteem support, expressions that convey love and caring (emotional support) and respect and confidence in ability (esteem support), were considered as one category [106]. People with health concerns may express their disease feelings to mitigate their stress and appeal for sympathy [113], offer hope and a sense of empowerment to each other [114], and encourage peers’ engagement in medical care [115].

Network support content refers to expressions that communicate belonging to a group of people who have the same concerns or interest [106]: individual users use social media to build community with people who have similar health concerns or health goals [116], foster a group identity [116], enhance a sense of belonging [117], achieve shared health goals together [118], get connected with peer patients [119], and get involved in social events such as swimming lessons with peers [120]. Network support also empowered people with health concerns to influence health awareness at the societal level such as raising awareness of queer and trans issues [121].

People with health concerns may benefit from participating in online communities in several ways. First, they do not have to reveal personal identity and thus are free from the risk of being stigmatized when participating in online communities [107]. Second, online communities as an information channel supplement offline communication channels in terms of content and time. Peers may provide more information on treatment options not raised by health professionals because of time constraints [109]. Also, people can get answers and emotional support from online communities 24/7, while other social networks may not be able to provide such timely support [122]. Third, people have a sense of control over the help-seeking process when participating in online communities, which may fulfill patients’ need of autonomy and reduce treatment avoidance [123]. Last, engaging in online communities may benefit health outcomes; studies found that perceived social support predicted subjective well-being [124].

##### Track and Share Health Statuses or Activities

Social media enables users to track and share their health statuses or activities and view those of others in the community. The public can document and share their illness experiences such as a breast cancer journey [125] or experience with chronic disease [126], achievements in health behavior change [127], and fitness activities [128] such as cycling and walking [129]. By self-tracking and sharing their physical activities, users can build networks with other users and discuss their activity performances [129]. Writing about and sharing their illness experiences may help patients cope with illness [125] and achieve health goals [126]. Other users may also benefit from such sharing as they can easily find peers who have similar experience to get advice on lifestyle changes and discuss treatment options [130].

## Discussion

We reviewed articles published between 2006 and 2020 and identified 10 uses of social media for health purposes. The review found new social media uses for health purposes that emerged after 2013 as well as research gaps that need future research. The following sections will discuss the new uses and gaps.

### New Social Media Uses for Health Purposes

Several new usages have emerged since 2013 compared with the usages summarized by Moorhead et al [12]. First, recent studies have begun to explore social media use for health purposes beyond the scope of patients, the general public, and health professionals. Studies have paid attention to social media use by health institutions including government agencies such as US federal, state, and local health departments [3,28,131] and nongovernmental health organizations [62]. Social media use identified in Moorhead et al [12] mostly benefits individuals who are experiencing illness or with the need for health information, while the use of social media by government agencies may benefit the whole society, especially during disease outbreaks. These articles also mentioned the limitations of government agencies using social media for health purposes such as lack of conversation with the public [131] and a disconnect between the health-related content generated by government agencies on social media and the actual health conditions that affect the population [2].

Second, studies in recent years have started to pay attention to social media use by health professionals and researchers to advance their practices and research. Social media is not only a communication channel that facilitates their conversation with patients [85] but also a tool for professional development [132] and health-related research [75]. Social media can provide opportunities for professional development from job searches to learning about trending research findings [77,80]. In addition, although studies have long been using social media for recruitment as a research tool, research efforts have been made to investigate the effectiveness of using social media for recruitment in recent years (ie, 2019-2020) and found social media promising as a recruitment tool [75].

Third, studies in recent years have found that social media can be used for social mobilization. Social media provides new opportunities for nongovernmental organizations to build community, mobilize resources, and extend discourse about sensitive issues beyond mainstream media coverage [47]. Although social mobilization was often initiated by nonprofit health organizations, people with heath concerns can form a community and collectively use social media to raise awareness of a disease or minority population. For example, patients with brain injuries can use social media to increase awareness of brain injury in society [133]. Social media was also used by LGBTQIA+ youth in the United States to increase the visibility of that population’s voice and promote civic engagement on public issues related to the population [121].

Last, as social media incorporates more functions such as sending reminders, registering for events, and linking payment methods, social media becomes useful in facilitating offline health-related services and events such as making appointments and providing visiting guides [90]. In this sense, social media not only serves as a communication tool but also a tool for medical service and administration. However, this emerging usage may not be applicable to all social media platforms, as different platforms may incorporate functions not suitable for administrative uses. Moreover, this usage poses challenges to health organizations as they need to concern about the privacy issue and the need of additional workforces to manage their social media accounts [90].

### Research Gaps in Social Media Uses for Health Purposes

There are several gaps existing in this research on using social media for health purposes. First, future studies should explore and test strategies to motivate engagement based on evidence of audience segmentation. There have been research efforts devoted to examining the audience segments on social media based on network characteristics in a health campaign [58], the extent to which users produce and consume health-related user-generated content [134], and users’ motivation for participating in an online community [135]. Although these studies on audience segmentation provide implications on strategies to engage different types of audiences, we need more studies to formally test the effectiveness of strategies derived from audience segmentation research.

In addition, future research should develop a dynamic audience segment–detecting tool that can monitor audience characteristics and integrate these characteristics to predict audience segments on social media. Multiple factors may collaboratively determine audience types such as offline support [134], sentiment about the health issue [58], illness stage, need for support [135], and availability of spare time [136]. Some of these factors may change over time resulting in a transitioning of audience types [135]. As a result, changes in social media users’ health status and social environments may lead to changes in their engagement pattern on social media. By predicting the change in user engagement patterns, practitioners can develop tailored engagement strategies for different types of social media users and adapt the strategies according to individual changes as an intervention goes on.

Second, more studies are needed exploring the relative impact of different applications of social media in health interventions on health outcomes, a research gap identified by Moorhead et al [12] that remains unresolved. Although the general impact of integrating social media in health interventions on promoting health knowledge and behavior is promising, it is unclear if different uses of social media in health interventions have different impacts. Social media can be integrated into an intervention in multiple ways and used for peer discussion with or without discussion leaders [137], conversations between participants and health professionals [138], delivering real-time and adaptive intervention messages [139] or regular intervention messages [45], delivering information in texts or more attractive formats such as video and infographics [138], intervention monitors to acknowledge achievements in participant health behavior changes [51], or participants to share their achievements with each other [127], etc. Using social media in these different manners may result in different effects on participant health behaviors and outcomes. For example, Garett et al [140] found that participants in the intervention group (ie, a Facebook group with peer leaders) acquired more knowledge about sexual health services and felt more comfortable with connecting with peers than those in the control group (ie, a Facebook group without peer leaders). Experimental studies and meta-analyses may be helpful to examine if and how using social media in an intervention in different ways affect health behavior change differently.

Third, social media enables users with similar health issues to develop health-related identities in a community. However, under which conditions identity building leads to positive or negative health behaviors and health outcomes is unclear [141]. Although developing identities may foster mutual support and decrease a sense of isolation [116], building an identity with a certain patient group can reinforce negative health behaviors such as disordered eating behaviors [142], and being part of a healthy community may pose pressures for users to consistently present an optimal identity as a healthy role model under persistent self- and community surveillance [128]. Researchers should explore approaches to using the identity-building function of social media to improve health outcomes and avoid negative impacts.

Last, privacy concerns were raised in articles on using social media for various health purposes including disease surveillance [21], health interventions [60], participation in online communities [143], and professionals communicating with colleagues and patients [76]. Researchers have consistently called for efforts to address privacy concerns related to using social media for health purposes [12,61,144]. Indeed, research effort has been made such as developing models to filter patients and caregivers and match them with trusted peer patients and caregivers in a privacy-preserving way [85] and developing social media privacy guide for health professionals [145]. However, gaps still exist in research and practice such as lacking an official guideline about privacy issues related to using social media in health research recruitment [73,146], lacking an approach to guarantee online informed consent [140], and researchers and potential participants lacking the awareness of the privacy risks of social media research recruitment [146]. Future studies should explore research methods and form practice guidelines that can address privacy issues associated with using social media for health research, intervention, and patient-doctor interaction.

### Limitations

The review has several limitations. First, the review does not include conference papers, dissertations, and grey literature. However, given the large number of peer-reviewed journal articles included in the review and overlaps on social media uses identified in the articles, we expect that there will not be many other social media uses left unidentified by the review.

Second, we only searched keywords in abstracts instead of full texts. This may render the review missing articles that mentioned social media uses for health purposes only in the main text and not in the abstract. Indeed, some articles were not included in this review but were included in previous reviews; all articles included in this study were published between 2016 and 2020, while the previous review conducted by Moorhead and colleagues [12] included articles published before 2013. However, although the scope of this review may be limited due to the restrictive search strategy, the uses identified in the review cover most social media uses found by Moorhead and colleagues [12], meaning this study provides a comprehensive overview of the social media uses identified in existing literature. In addition, this review contributes to the literature by identifying social media uses for health purposes emerging after 2013.

### Conclusions

This review summarized 10 social media uses for health purposes identified in previous literature and categorized the social media uses in terms of user types. Public health practitioners and organizations may use the summary as a starting point to explore applying social media in their daily practice. In addition, the review contributes to the literature by identifying research gaps in social media use for health purposes, providing guidance for future research in this area.

## Abbreviations

LGBTQIA+: lesbian, gay, bisexual, transgender, queer, intersex, asexual
PRISMA: Preferred Reporting Items for Systematic Reviews and Meta-analyses
